# A Prognostic Pyroptosis-Related lncRNAs Risk Model Correlates With the Immune Microenvironment in Colon Adenocarcinoma

**DOI:** 10.3389/fcell.2021.811734

**Published:** 2021-12-13

**Authors:** Fada Xia, Yuanliang Yan, Cong Shen

**Affiliations:** ^1^ Center for Molecular Medicine, Xiangya Hospital, Key Laboratory of Molecular Radiation Oncology of Hunan Province, Central South University, Changsha, China; ^2^ National Clinical Research Center for Geriatric Disorders, Xiangya Hospital, Central South University, Changsha, China

**Keywords:** pyroptosis, lncRNA, colon adenocarcinoma (COAD), immune microenvironment, prognosis

## Abstract

Recent studies have indicated that long non-coding RNAs (lncRNAs) may participate in the regulation of tumor cell proptosis. However, the connection between lncRNA expression and pyroptosis remains unclear in colon adenocarcinoma (COAD). This study aims to explore and establish a prognostic signature of COAD based on the pyroptosis-related lncRNAs. We identify 15 prognostic pyroptosis-related lncRNAs (ZNF667-AS1, OIP5-AS1, AL118506.1, AF117829.1, POC1B-AS1, CCDC18-AS1, THUMPD3-AS1, FLNB-AS1, SNHG11, HCG18, AL021707.2, UGDH-AS1, LINC00641, FGD5-AS1 and AC245452.1) from the TCGA-COAD dataset and use them to construct the risk model. After then, this pyroptosis-related lncRNA signature is validated in patients from the GSE17536 dataset. The COAD patients are divided into low-risk and high-risk groups by setting the median risk score as the cut-off point and represented differences in the immune microenvironment. Hence, we construct the immune risk model based on the infiltration levels of ssGSEA immune cells. Interestingly, the risk model and immune risk model are both independent prognostic risk factors. Therefore, a nomogram combined risk score, immune risk score with clinical information which is meaningful in univariate and multivariate Cox regression analysis is established to predict the overall survival (OS) of COAD patients. In general, the signature consisted of 15 pyroptosis-related lncRNAs and was proved to be associated with the immune landscape of COAD patients.

## Introduction

Colorectal cancer (CRC) is one of the most frequent malignancies and the third leading cause of cancer-related death globally. Based on the pathologic classification, colon adenocarcinoma (COAD) is the most common subtype of colon cancer (Sui et al., 2020; Yu et al., 2020). In recent years, the incidence of COAD has been rising among adolescents. Currently, the 5-years overall survival (OS) rate of COAD patients without distant metastasis has a good prognosis, but that of patients with metastases is less than 15% (Almatroudi, 2020; Gan et al., 2020). Although the AJCC TNM staging system as the prognostic indicator of COAD patients is continually updated, a significantly different prognosis still exists in patients with the same clinicopathologic characteristics (Lino-Silva et al., 2020; Ren et al., 2020). Therefore, identifying specific and sensitive prognostic biomarkers is imperative to predict the outcomes of COAD patients.

Pyroptosis is a newly-found programmed cell death (PCD) dependent on inflammasome, characterized by pore formation in the plasma membrane, causing cell swelling and rupture, as well as the escape of proinflammatory contents (Kolbrink et al., 2020). Pyroptosis is a double-edged sword, playing a key role in resisting bacterial and viral infections. Meanwhile, it is directly associated with tumor development (Li et al., 2020; Shi et al., 2020). Additionally, pyroptosis is related to the antitumor response by cross-linking between immunity and the cancer microenvironment (Xiang et al., 2020). Hence, it gives us a novel direction for the therapeutic targets of tumors. In recent years, some research has revealed the potential linkages between the pyroptosis-related pathways and COAD (Gong et al., 2021). However, the biological mechanism for the pyroptosis influence on tumor progression of COAD is still unclear.

LncRNA (long non-coding RNA) refers to a type of RNA that is more than 200 nt in length and does not encode proteins but mediates gene expression at multiple levels (Lin et al., 2018). Numerous studies have identified that lncRNA is indispensable in tumorigenesis and development and even alters the tumor properties, leading to tumor microenvironment differences (Mercer et al., 2009; Qiu et al., 2021). Furthermore, the underlying mechanism between pyroptosis and lncRNAs remains to be deciphered, considering few studies elucidate the role of pyroptosis-related lncRNAs in predicting COAD prognosis and the association between those lncRNAs and the immune microenvironment. Thus, it is feasible to construct a risk model composed of pyroptosis-related lncRNAs, which can help us to precisely predict the prognosis of COAD patients and develop immunotherapies in the future.

**FIGURE 1:**
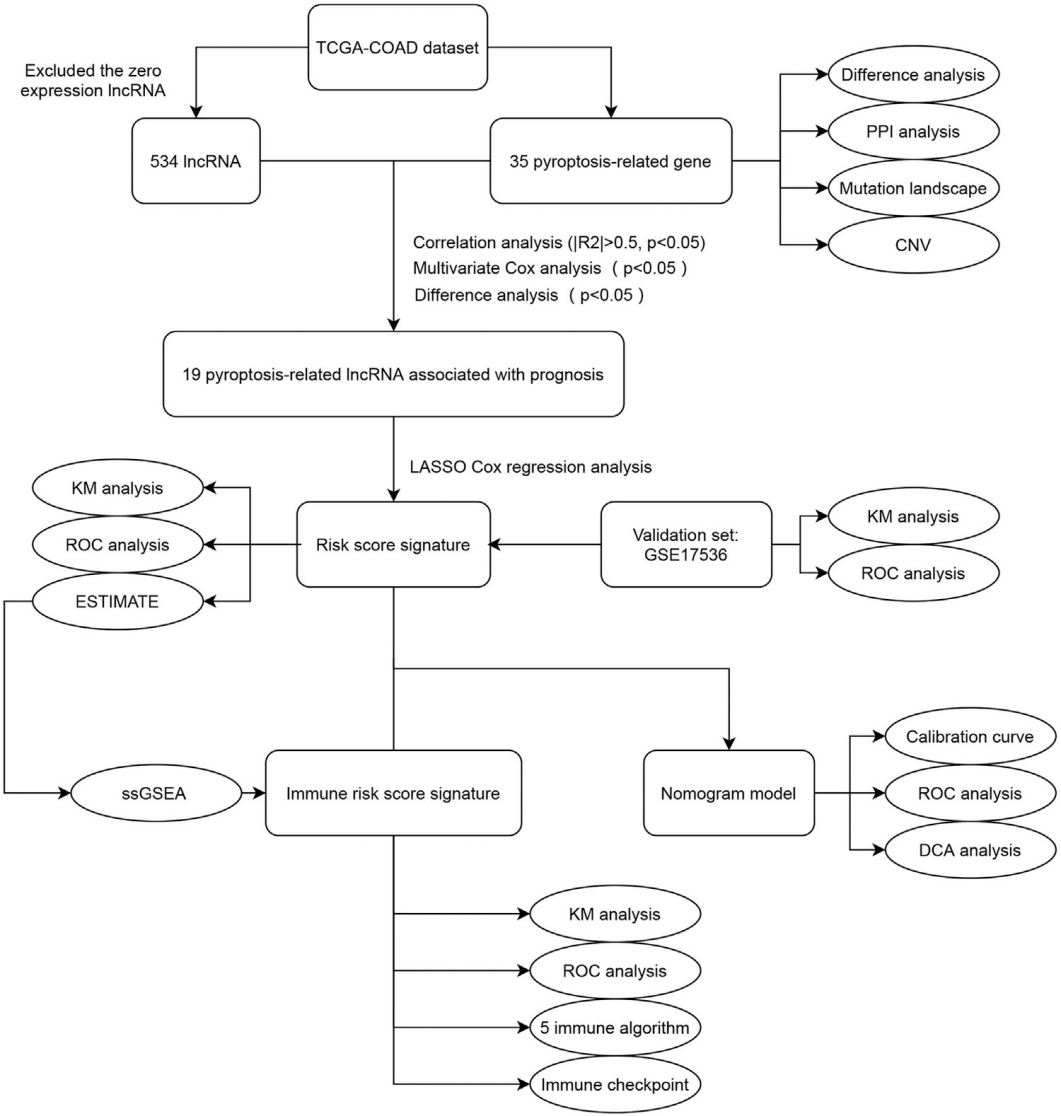
The flowchart of this study. TCGA, The Cancer Genome Atlas; COAD, colon adenocarcinoma; PPI, protein-protein interaction; CNV, copy number variation; KM, Kaplan-Meier; ROC, receiver operating characteristic; ESTIMATE, Estimation of STromal and Immune cells in MAlignant Tumor tissues using Expression data; ssGSEA, single sample Gene Set Enrichment Analysis; DCA, decision curve analysis.

In this study, we systematically explored the relationship between pyroptosis-related lncRNAs and probed into clinicopathological information and the tumor immune landscape of COAD. We initially screened differentially expressed pyroptosis-related lncRNAs to build a prognostic risk model. Based on the median cut-off of risk score, we divided COAD patients into high-risk and low-risk groups. We also employed an external cohort to validate the prognostic predictive ability of the risk model. Then, the immune state of the two groups was assessed; accordingly, an immune risk model was constructed. To the end, we combined predictable clinicopathological features, risk score and immune risk score to construct an efficient nomogram to predict the survival rate of COAD patients. The detailed flowchart is shown in Figure 1. This study provides a new reference to predict the prognosis of COAD patients and identify personalized treatment strategies.

## Materials and Methods

### Datasets and Clinical Data

The RNA-seq dataset of TCGA-COAD with matching clinical information was downloaded from the UCSC Xena browser (https://xena.ucsc.edu/). The gene annotation files (version GRCh38.99) were downloaded from the Ensembl genome browser (http://asia.ensembl.org/index.html). Patients without exact clinical information were excluded for the subsequent analysis. We got 429 COAD samples and 37 paired normal samples as the training cohort (Table 1). The GEO datasets (https://www.ncbi.nlm.nih.gov/geo/) with the accession number was GSE17536 (Smith et al., 2010), were used to verify the prognostic model. We discarded the cases with missing information in this analysis. The entire process was analyzed by R (version 4.0.3).

**TABLE 1:** Characteristics of TCGA-COAD dataset.

Characteristics	Tumor (N = 429)	Normal (N = 37)		Total (N = 466)	*p* Value	FDR
					
Gender					0.99	1
Female	202 (43.35%)		18 (3.86%)	220 (47.21%)		
Male	227 (48.71%)		19 (4.08%)	246 (52.79%)		
					
Age						
Mean ± SD	66.70 ± 12.77		69.70 ± 13.46	66.94 ± 12.84		
Median [min-max]	69.00 [31.00,90.00]		74.00 [40.00,90.00]	69.00 [31.00,90.00]		
					
Disease type					0.11	0.66
Adenocarcinoma	372 (79.83%)		28 (6.01%)	400 (85.84%)		
Mucinous adenocarcinoma	57 (12.23%)		9 (1.93%)	66 (14.16%)		
					
Pathologic stage					0.18	0.88
Stage Ⅰ	74 (15.88%)		4 (0.86%)	78 (16.74%)		
Stage Ⅱ	170 (36.48%)		20 (4.29%)	190 (40.77%)		
Stage Ⅲ	123 (26.39%)		6 (1.29%)	129 (27.68%)		
Stage Ⅳ	62 (13.30%)		7 (1.50%)	69 (14.81%)		
					
T stage					0.48	1
T1	9 (1.93%)		0 (0.0e+0%)	9 (1.93%)		
T2	75 (16.09%)		4 (0.86%)	79 (16.95%)		
T3	297 (63.73%)		27 (5.79%)	324 (69.53%)		
T4	48 (10.30%)		6 (1.29%)	54 (11.59%)		
					
N stage					0.40	1
N0	253 (54.29%)		26 (5.58%)	279 (59.87%)		
N1	99 (21.24%)		6 (1.29%)	105 (22.53%)		
N2	77 (16.52%)		5 (1.07%)	82 (17.60%)		
					
M stage					0.62	1
M0	367 (78.76%)		30 (6.44%)	397 (85.19%)		
M1	62 (13.30%)		7 (1.50%)	69 (14.81%)		

FDR, false discovery rate; SD, standard deviation

### Identification of Pyroptosis-Related Genes

Based on previous studies (Ye et al., 2021), we extracted a total of 35 pyroptosis-related genes for differentially expressed analysis; 26 of them were differentially expressed between normal and tumor samples through the rank sum test. The significance differences were indicated as follows: *p* < 0.05 as *, *p* < 0.01 as **, and *p* < 0.001 as ***. The gene circular map showed mutated pyroptosis-related genes. The t-SNE was used to distinguish normal and tumor samples; the correlation analysis was used to discover interrelation based on the expression of pyroptosis-related genes. The protein-protein interaction (PPI) was analyzed with STRING (https://string-db.org/) by setting the minimum interaction sources as 0.400 (Szklarczyk et al., 2021).

### Identification of Pyroptosis-Related LncRNAs

We removed lncRNAs with zero expression after annotation and then acquired 534 lncRNAs for subsequent correlation analysis. Then, pyroptosis-related lncRNAs satisfied the correlation coefficient |*R* ^2^| > 0.5 and *p* < 0.05 by Pearson correlation analysis. The prognostic values of pyroptosis-related lncRNAs were assessed by multivariate Cox analysis (*p* < 0.05), and difference analysis was further conducted (*p* < 0.05). Finally, 19 pyroptosis-related lncRNAs were obtained to construct a prognostic risk signature.

### Development and Validation of Prognostic Pyroptosis-Related LncRNAs Signature

Lasso Cox regression analysis was used to build a prognostic model to avoid overfitting (Tibshirani, 1997). We got 15 pyroptosis-related lncRNAs to construct the model by setting the λ parameter as 0.009 through the “glmnet” R package. Each lncRNAs with coefficients contributed differently to prognosis, and each patients’ risk scores were calculated by the following formula: risk score = $\overset{n}{\underset{i=0}{\sum }}expr\;i*coef\;i$ . We divided COAD patients into the low-risk and high-risk groups by setting the median risk score as the cut-off point. To compare the OS difference between the low- and high-risk groups, we used the “survminer” R package. To investigate the predictive ability of the prognostic model over time, we employed the “TimeROC” R package to show the time-dependent ROC curve. In order to confirm the broad applicability of the prognostic model, we introduced a validation set: GSE17536. The predictive ability of the model was repeatedly validated by the methods mentioned above.

### Development and Validation of Immune Infiltration Signature

ESTIMATE is an algorithm for calculating tumor purity and the percentage of infiltrating stromal/immune cells in tumor samples (Yoshihara et al., 2013). After comparing the results of ESTIMATE between the low- and high-risk groups, we used ssGSEA (single-sample gene set enrichment analysis) to evaluate the infiltration of 28 immune cells. Also, using Lasso Cox regression analysis, an immune prognostic model was built by infiltrating scores of three immune cells. The Kaplan-Meier (K-M) survival analysis and time-dependent ROC curve were performed to validate the predictive ability of the immune prognostic model. We employed more immune infiltration algorithms (CIBERSOFT, EPIC, IPS, MCP counter and TIMER) to assess the difference of immune microenvironment between the two immune-risk groups (Becht et al., 2016; Li et al., 2016; Charoentong et al., 2017; Racle et al., 2017; Newman et al., 2019). Immune checkpoint blockade (ICB) response was predicted by ImmuCellAI (http://bioinfo.life.hust.edu.cn/ImmuCellAI#!/) to check the response of ICB in tumor samples (Miao et al., 2020).

### Construction of Nomogram

The nomogram was based on the multivariate regression analysis of clinicopathological information, risk score and immune risk score. We used the “rms” R package to build the nomogram to predict the 1-, 3- and 5-years survival rates of COAD patients. The ROC curve and the calibration curve were utilized to estimate the suitability of clinical use. The alluvial plot was used to describe the shunt of each patient with different clinicopathological features. The DCA (decision curve analysis) was used to evaluate the clinical utility of the nomogram and help clinicians make decisions.

### Statistical Analysis

R version 4.0.3 and various R packages were used to perform all statistical analyses. The chi-squared test was used to compare the constituent radio of two subgroups. The “Rtsne” R package was used to analyze *t*-SNE. The Pearson correlation analysis was used to evaluate the correlation between two variables. Univariate and multivariate Cox regression analysis was run by the “survival” R package. Hazard ratio (HR) > 1 was considered as a risk factor, while HR > 1 was considered as a protective factor. The “GSVA” R package was utilized to perform the ssGSEA. The distribution of immune cell infiltration and immune check-point between the two groups was analyzed by the nonparametric test. The “rmda” R package was used to create the DCA curve.

## Results

### Survey of Different Expression and Genetic Alterations of Pyroptosis-Related Genes in COAD

**FIGURE 2:**
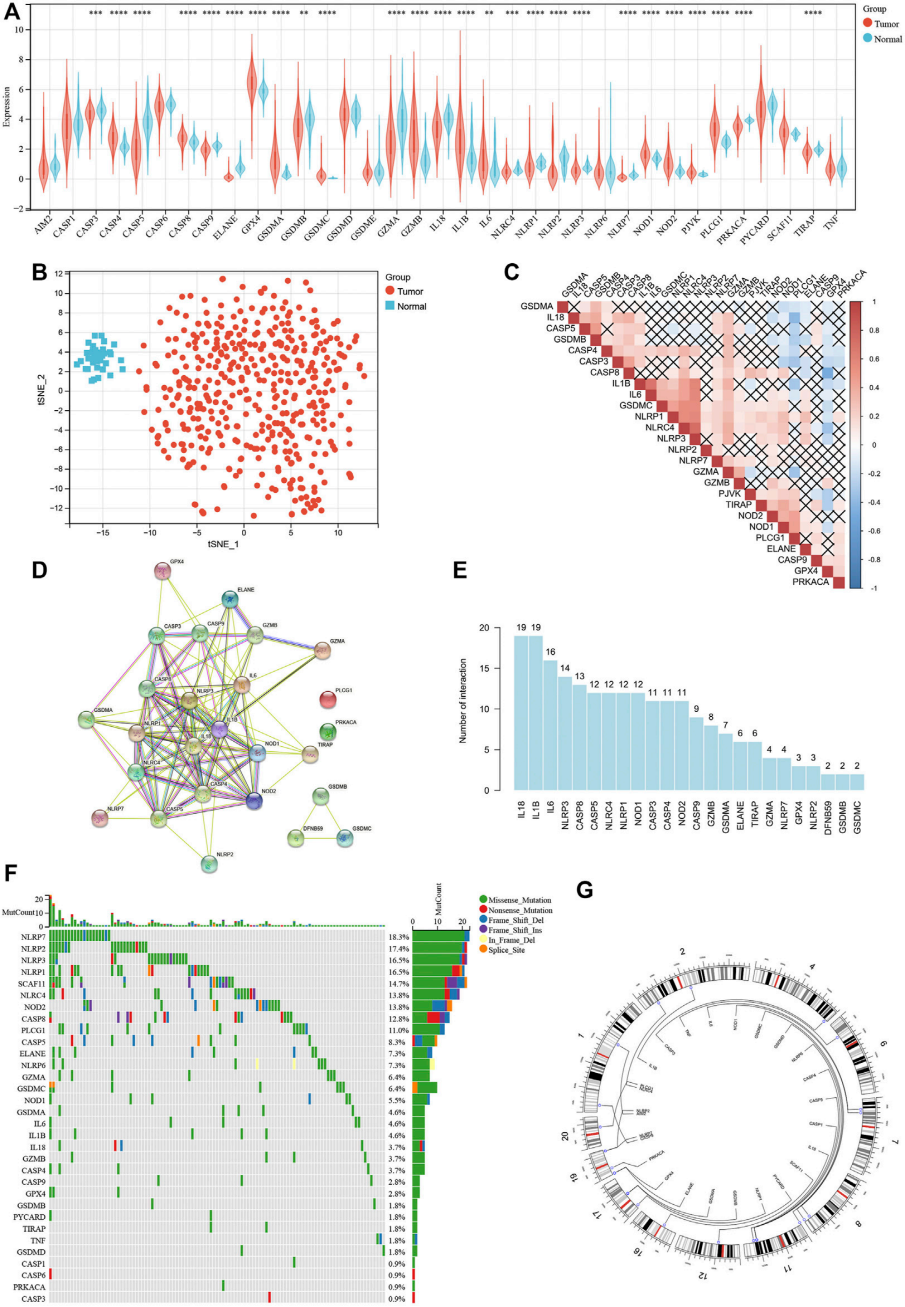
Survey of different expression and genetic alterations of pyroptosis-related genes in COAD. **(A)** Boxplot of the expression levels of 35 pyroptosis-related genes between tumor and normal samples. **(B)** *t*-SNE showing the distribution between tumor and normal samples. **(C)** Correlation among 16 differently expressed pyroptosis-related genes. **(D)** PPI network among 16 differently expressed pyroptosis-related genes. **(E)** Histogram of interaction among 16 differently expressed pyroptosis-related genes. **(F)** Mutation landscape of 399 COAD patients. Waterfall plot showing the mutation information of each pyroptosis-related gene. **(G)** The location of each pyroptosis-related gene on the chromosome. *p < 0.05; **p < 0.01; ***p < 0.001; ****p < 0.0001.

To find differently expressed pyroptosis-related genes in COAD, expression levels of 35 pyroptosis-related genes were compared between 429 tumor samples and 37 paired normal samples, identifying 26 genes representing significantly different expression (*p* < 0.05). Among them, 11 genes were upregulated, while the other 15 genes were downregulated in tumor samples (Figure 2A). Based on 26 differently expressed pyroptosis-related genes, t-SNE visualized the distribution between tumor and normal samples (Figure 2B). Also, correlation analysis (Figure 2C) and PPI network (Figure 2D) were performed to show the complex interactive relationship among the 26 pyroptosis-related genes. As shown in the histogram of interaction, IL-18 and IL-1β had the largest number of interactions (Figure 2E). At the level of genetic mutations, about 111 of 399 (approximately 27.82%) tumor samples represented mutations of pyroptosis-related genes, and NLRP7 accounted for the most (Figure 2F). Then, we mapped these mutated pyroptosis-related genes on the chromosome (Figure 2G). The alterations of pyroptosis-related genes might play an important role in COAD formation.

### Identification of Pyroptosis-Related LncRNAs With Prognostic Value

Based on the TCGA-COAD RNA-seq dataset, 534 lncRNAs were included for further research by screening out lncRNAs with zero expression. Then, the correlation analysis between 26 pyroptosis-related genes and 534 lncRNAs was constructed under the specific conditions that Pearson correlation coefficient |*R* ^2^| > 0.5 and *p* < 0.05. After taking the 118 pyroptosis-related lncRNAs into multivariate Cox analysis (*p* < 0.05) and difference analysis (*p* < 0.05), we identified that 19 of them were associated with prognosis (all the results of multivariate Cox analysis were shown in Supplementary Material).

**FIGURE 3:**
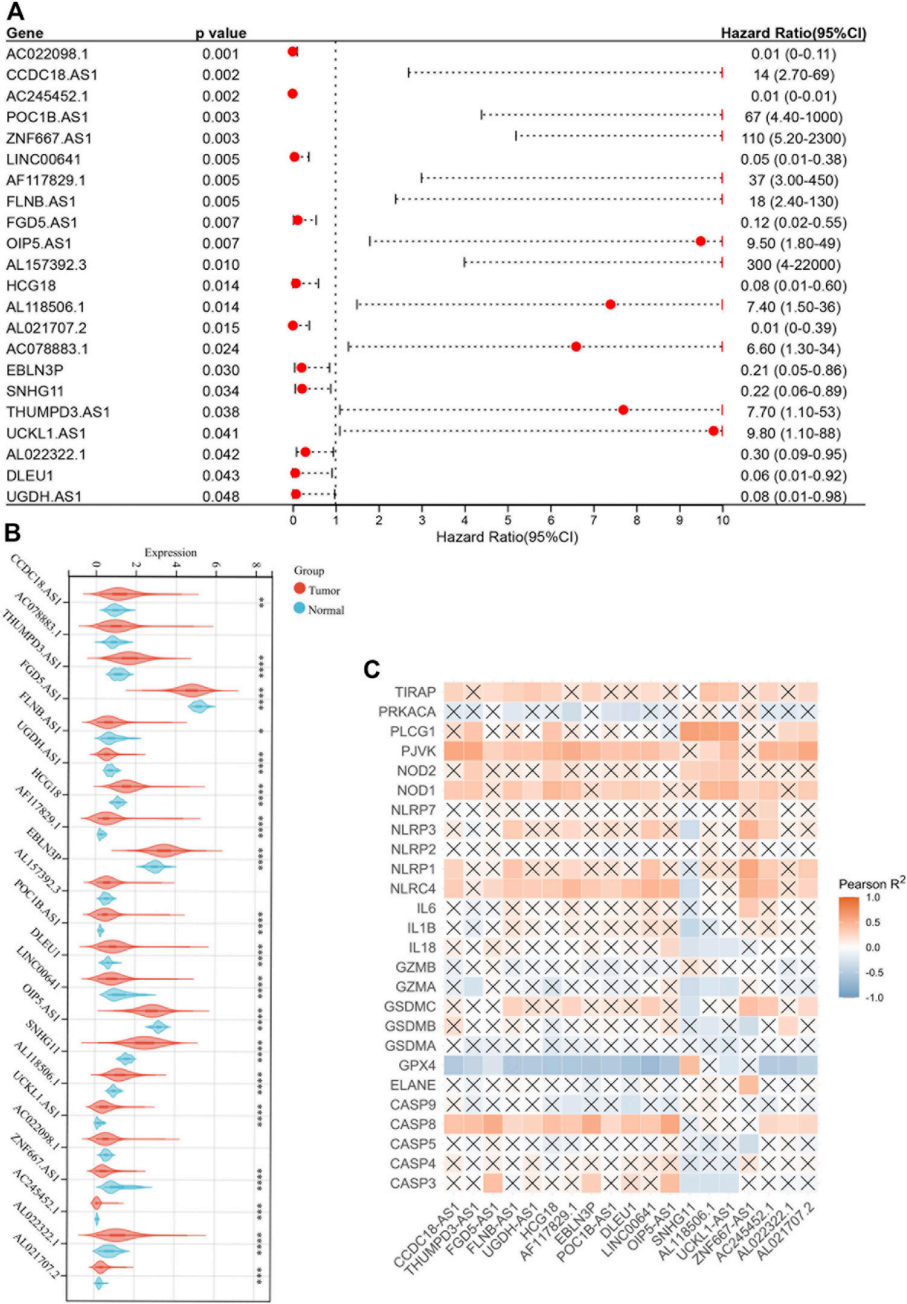
Identification of pyroptosis-related lncRNAs with prognostic value. **(A)** Forest plot of 22 pyroptosis-related lncRNAs after multivariate Cox regression analysis (*p* < 0.05). **(B)** Boxplot of 22 pyroptosis-related lncRNAs. **(C)** Correlation between 22 pyroptosis-related lncRNAs and 26 pyroptosis-related genes. *p < 0.05; **p < 0.01; ***p < 0.001; ****p < 0.0001.

The forest plot showed 22 pyroptosis-related lncRNAs with prognostic significance after multivariate Cox analysis (Figure 3A). The correlation heatmap was plotted according to the difference analysis results (Figure 3B). Moreover, GPX4 was negatively correlated with most pyroptosis-related lncRNAs, while PJVK, NOD1, NLRC4 and CASP8 were positively correlated (Figure 3C).

### Construction and Validation of a Risk Model Based on Pyroptosis-Related LncRNAs

**FIGURE 4:**
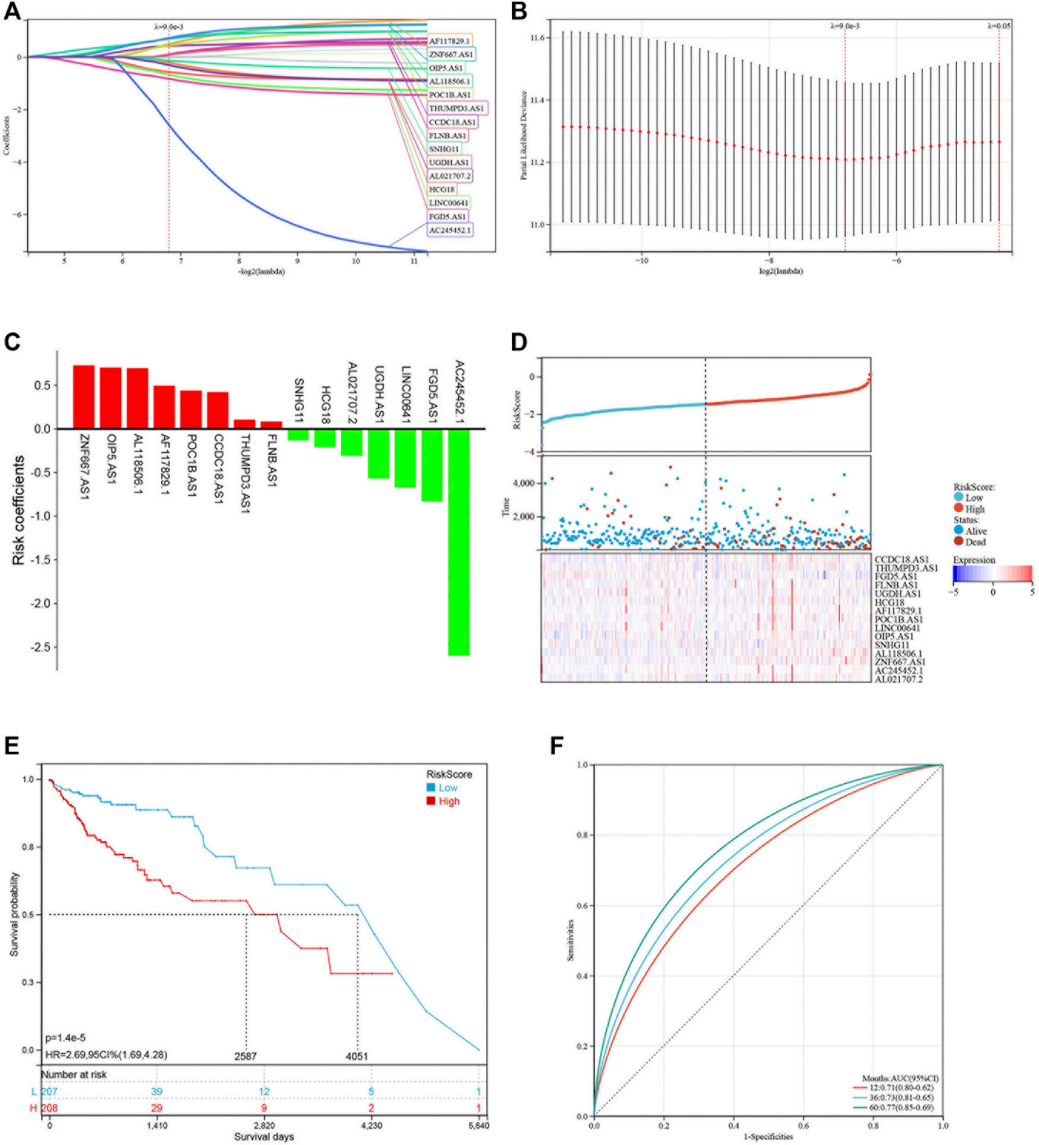
Construction of the risk model based on pyroptosis-related lncRNAs. **(A,B)** LASSO regression of 16 pyroptosis-related lncRNAs. **(C)** LASSO coefficients of 16 pyroptosis-related lncRNAs. **(D)** Distribution of risk score and survival status of COAD patients. Heatmap showing the expression levels of 16 pyroptosis-related lncRNAs. **(E)** Kaplan-Meier curve showing the survival difference between low-risk and high-risk groups. **(F)** ROC curve of the risk score in predicting 1-, 3-, and 5-years OS.

**FIGURE 5:**
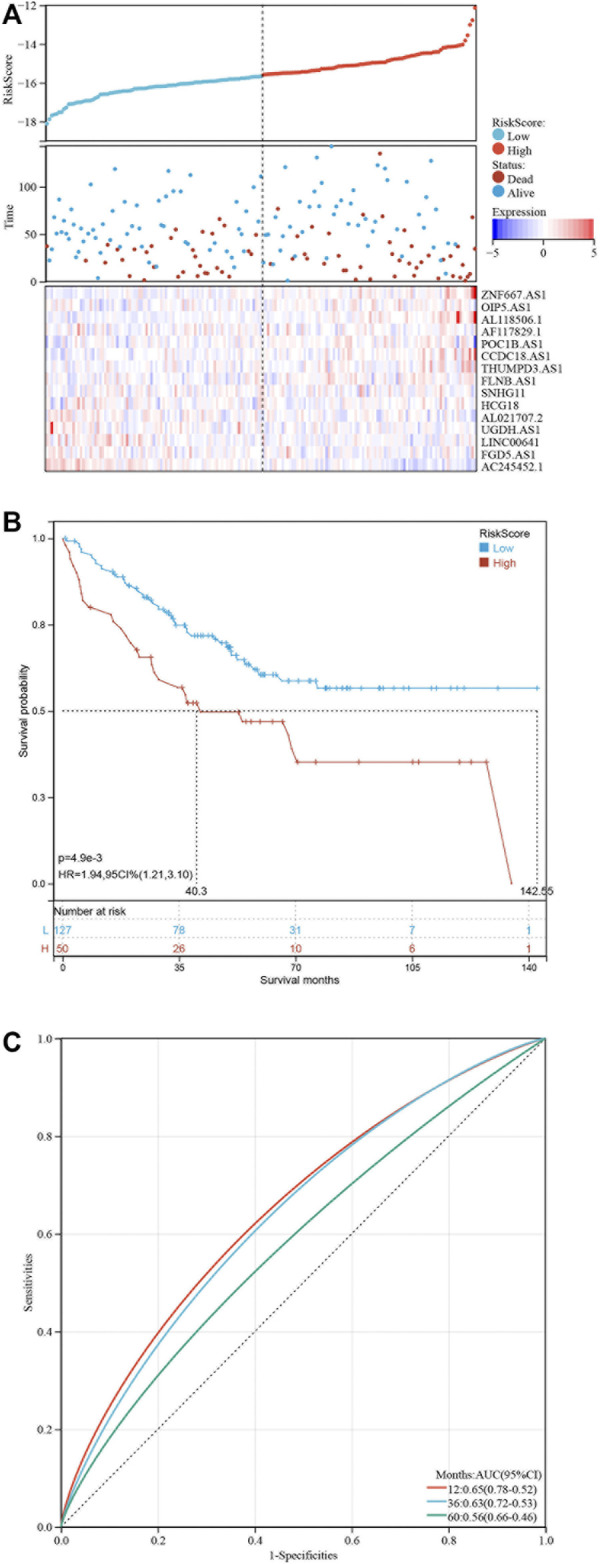
Validation of the risk model at GSE17536. **(A)** Distribution of risk score and survival status of COAD patients. Heatmap showing the expression levels of 16 pyroptosis-related lncRNAs. **(B)** Kaplan-Meier curve showing the survival difference between low-risk and high-risk groups. **(C)** ROC curves of the risk score in predicting 1-, 3-, and 5-years OS.

Based on the 19 pyroptosis-related lncRNAs identified above, a risk model was created by Lasso Cox regreesion analysis to calculate the risk score of each patient (Figures 4A,B). By setting the compression parameter λ = 0.009, 15 lncRNAs were enrolled to build the risk model with each coefficient (Figure 4C). The risk score = (ZNF667-AS1) × (0.728,496) + (OIP5-AS1) × (0.704,723) + (AL118506.1) × (0.695,672) + (AF117829.1) × (0.495,352) + (POC1B-AS1) × (0.441,168) + (CCDC18-AS1) × (0.422,851) + (THUMPD3-AS1) × (0.105,734) + (FLNB-AS1) × (0.086465) + (SNHG11) × (−0.132,084) + (HCG18) × (-0.211,577) + (AL021707.2) × (−0.310,627) + (UGDH-AS1) × (−0.569,069) + (LINC00641) × (−0.674,802) + (FGD5-AS1) × (−0.831,751) + (AC245452.1) × (−2.598,539). Subsequently, we divided 429 COAD patients into high-risk and low-risk groups by setting the median risk score as the cut-off point (Figure 4D). As the risk score rose, the death numbers increased, and the survival time decreased. As shown by the Kaplan-Meier curve, COAD patients with high-risk scores had a poorer prognosis than those with low-risk scores (Figure 4E). Furthermore, the ROC curve demonstrated the predictive ability of the risk score in terms of prognosis; the areas under the curves (AUC) of 1, 3 and 5 years were 0.71, 0.73 and 0.77, respectively (Figure 4F). For validation in external datasets, we employed GSE17536 as the validation set and repeated the above analysis (Figure 5A). The same results were obtained that patients with low-risk scores had a better prognosis (Figure 5B). The risk score had a good prognostic predictive ability with the AUC of 0.65, 0.63 and 0.56 in 1, 3 and 5 years (Figure 5C).

### Construction of an Immune Risk Model Based on Immune Cell Infiltration

**FIGURE 6:**
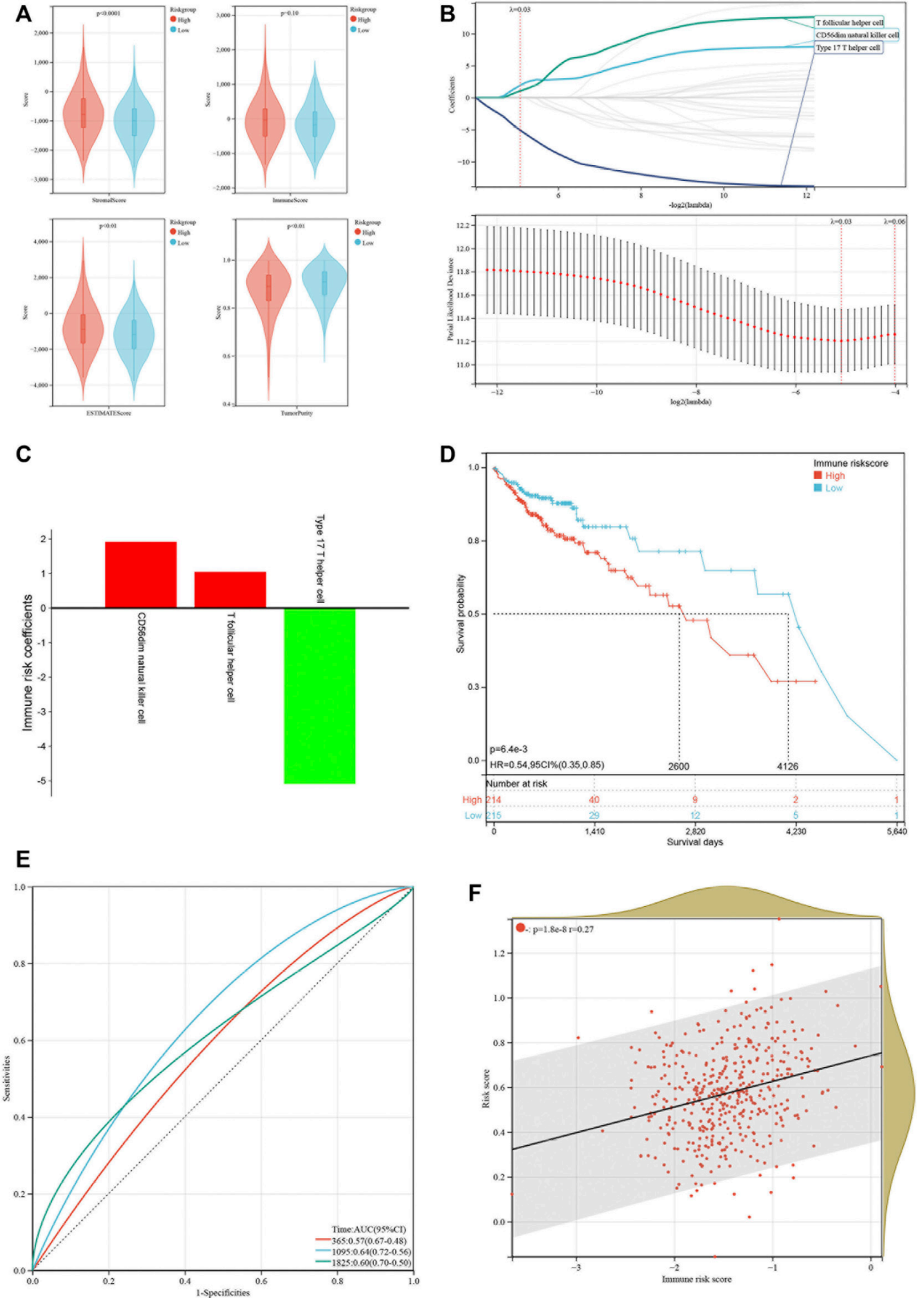
Construction of an immune risk model based on immune cell infiltration. **(A)** Comparison of immune score, stromal score, ESTIMATE score and tumor purity between low-risk and high-risk groups. **(B)** LASSO coefficients of three immune cells. (C) LASSO coefficients of three immune cells. **(D)** Kaplan-Meier curve showing the survival difference between low-immune-risk and high-immune-risk groups. **(E)** ROC curves of the immune risk score in predicting 1-, 3-, and 5-years OS. **(F)** Linear relationship of risk score and immune risk score.

**FIGURE 7:**
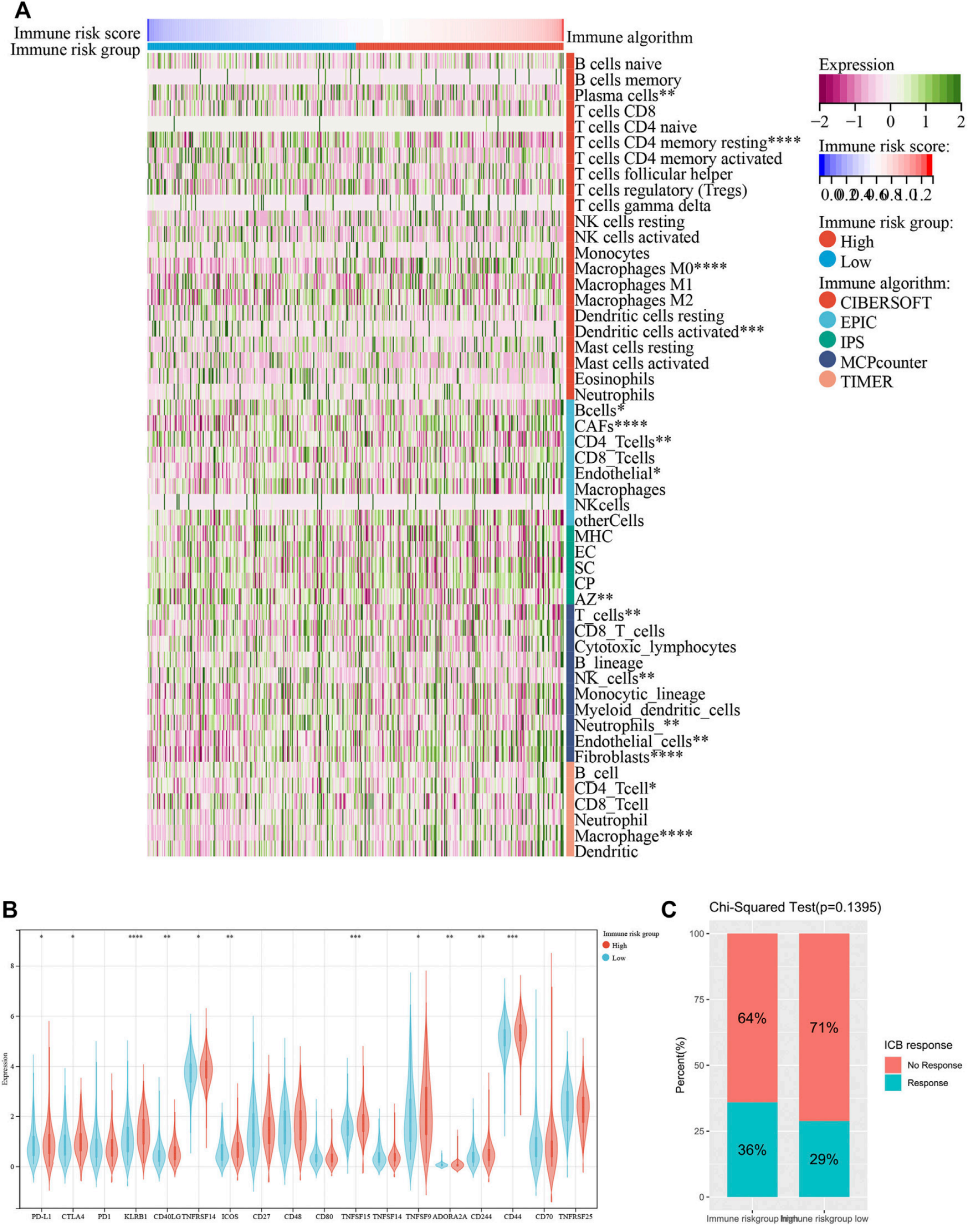
Other immune algorithms employed in comparison between low-immune-risk and high-immune-risk groups. **(A)** Heatmap showing the infiltration levels of immune cells under CIBERSOFT, EPIC, IPS, MCP counter and TIMER algorithms. **(B)** Boxplot showing the expression levels of immune checkpoint between low-immune-risk and high-immune-risk groups. **(C)** Result of Chi-squared test of ICB response between low-immune-risk and high-immune-risk groups. ICB, immune checkpoint blockade. *p < 0.05; **p < 0.01; ***p < 0.001; ****p < 0.0001.

Emerging studies have pointed that immune-associated signaling pathways have important roles in cancer pathogenesis, including COAD (Xu et al., 2020; Zaiachuk et al., 2021). ESTIMATE was utilized to analyze the tumor immune microenvironment and score in each COAD sample. Interestingly, stromal score (*p* < 0.001), ESTIMATE score (*p* < 0.01) and immune score (*p* = 0.10) were higher in the high-risk group than in the low-risk group, while tumor purity (*p* < 0.01) represented contrary results (Figure 6A). To better dissect the immune microenvironment, we utilized the ssGSEA algorithm to derive the infiltration degree of 28 immune cells in tumors. Lasso Cox regression analysis was performed again, and three immune cells were selected with the parameter λ = 0.03 (Figure 6B). The immune risk score = (CD56dim natural killer cell) × (1.916,028) + (T follicular helper cell) × (1.049875) + (type 17 T helper cell) × (−5.093948) (Figure 6C). Based on the median immune risk score, COAD patients were divided into the high-immune-risk and low-immune-risk groups. Likewise, a high immune risk score indicated a poor prognosis (Figure 6D). The prognostic predictive ability of the immune risk score was measured by the AUC to be 0.57, 0.64, and 0.60 in 1, 3 and 5 years, respectively (Figure 6E). From the linear analysis, we found that the immune risk score was positively related to the risk score (r = 0.27 and *p* < 0.001) (Figure 6F). More immune algorithms were involved in demonstrating the immune microenvironment in multiple dimensions (Figure 7A). We further analyzed the expression levels of immune checkpoints between the two groups, and most of them were upregulated in the high-immune-risk group (Figure 7B). More patients in the high-immune-risk group (36%) responded to ICB than those in the low-immune-risk group (29%) (chi-squared test, *p* = 0.1395) (Figure 7C).

### Development of a Nomogram Incorporated With Clinicopathological Information

**FIGURE 8:**
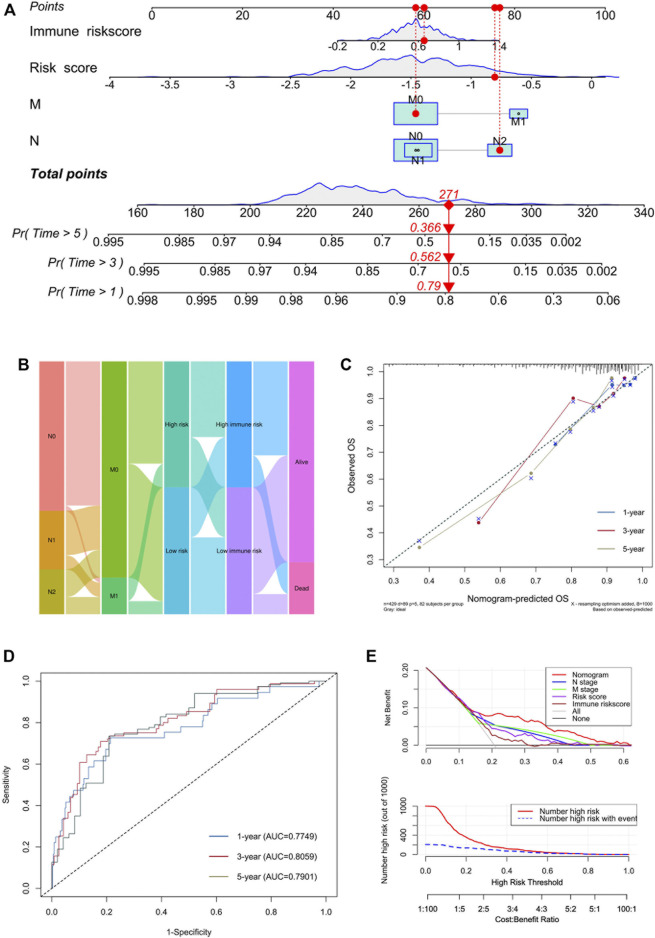
Development of a nomogram incorporated with clinicopathological information. **(A)** Nomogram based on N stage, M stage, risk score and immune risk score in predicting 1-, 3-, and 5-years OS. **(B)** Alluvial plot showing the outcome of each COAD patient. **(C)** Calibration curves of predicted and observed 1-, 3-, and 5-years OS. **(D)** ROC curves of nomogram in predicting 1-, 3-, and 5-years OS. **(E)** DCA analysis of nomogram and component factors.

To better expend the clinical application of the signature, a nomogram integrating with clinicopathological information was built. In the multivariate Cox analysis, we found that N stage, M stage, AJCC stage, risk score and immune risk score were significantly associated with prognosis, consistent with the result of univariate Cox analysis (Table 2). During the construction of the nomogram, the AJCC stage was the factor of overfitting, thus was excluded. By adding the fraction of each prognostic characteristic, each patient had a total point representing the survival rate of 1, 3 and 5 years (Figure 8A). Higher points were associated with a worse prognosis. The alluvial plot visualized the outcome of each patient (Figure 8B). The calibration curve showed that the OS predictive probability of nomogram in 1, 3 and 5 years was consistent with that of the actual observed OS (Figure 8C). Meanwhile, the nomogram represented convincing sensitivity and specificity in predicting clinical outcomes, exhibited by the ROC curve with the AUC of 0.77, 0.81, and 0.79 in 1, 3, and 5 years, respectively (Figure 8D). The DCA curve was used to solve the clinical utility problem of the nomogram. Compared with the traditional clinicopathological features, the nomogram had the optimum net benefit (NB) (Figure 8E).

**TABLE 2:** Univariate and multivariate Cox regression analysis in TCGA-COAD dataset.

Variables	N	Univariate analysis		Multivariate analysis	
		HR (95% CI)	*p* value	HR (95% CI)	*p* value
Age (years)	429	1.02 (1.00 1.04)	0.091		
					
Gender					
Female		1 (ref)			
Male		1.11 (0.73 1.70)	0.627		
					
T stage					
T1	9	1 (ref)			
T2	75	0.48 (0.05 4.68)	0.531		
T3	297	1.83 (0.25 13.21)	0.551		
T4	48	6.01 (0.80 45.04)	0.081		
					
N stage					
N0	253	1 (ref)		1 (ref)	
N1	99	1.70 (0.98 2.97)	0.06	0.29 (0.10 0.85)	0.023
N2	77	4.63 (2.82 7.58)	< 0.001	0.69 (0.26 1.86)	0.460
					
M stage					
M0	367	1 (ref)		1 (ref)	
M1	62	4.65 (2.98 7.24)	< 0.001	21.09 (4.91 90.64)	< 0.001
					
AJCC stage					
Stage Ⅰ	74	1 (ref)		1 (ref)	
Stage Ⅱ	170	2.42 (0.72 8.10)	0.153	2.15 (0.64 7.24)	0.215
Stage Ⅲ	123	4.77 (1.45 15.69)	0.010	9.60 (2.04 45.27)	0.004
Stage Ⅳ	62	13.72 (4.19 44.94)	< 0.001	NA	NA
					
Risk score	429	4.62 (2.93 7.26)	< 0.001	2.69 (1.24 5.82)	0.012
					
Immune risk score	429	10.95 (3.53 33.96)	< 0.001	3.68 (1.09 12.39)	0.036
HR, hazard ratio; CI, confidence interval; AJCC, American Joint Committee on Cancer					

## Discussion

Our study indicated that the prognostic signature based on the pyroptosis-related lncRNAs was a mighty predictive method for COAD patients. Meanwhile, the relation between risk score and immune microenvironment showed a potential contributing factor to poor prognosis. Hence, after integrating traditional clinicopathological information, risk score with the immune risk score, we constructed the unique prognostic evaluation system. The nomogram established a connection between the gene level and clinical prognosis. Factors affecting the prognosis of COAD patients were considered from multiple dimensions, forming a solid foundation for our clinical treatment.

Pyroptosis is a unique programmed cell death modality distinct from apoptosis. With research progressing, pyroptosis has been implicated in the activation of caspase-1, -4, -5, -11 and the maturation and release inflammatory cytokines IL-1β and IL-18 (Wang et al., 2019). As reported in numerous studies, pyroptosis is present in macrophages infected by HIV, dengue virus, adenovirus, etc. (Chen et al., 2012). In addition, pyroptosis has been reported to be involved in atherosclerosis and gouty arthritis. The present study identified the presence of inflammasomes in tumor cells that direct caspase-1 for pyroptosis, which may be an important node in the association of tumorigenesis and development. For example, the inflammasome NLRP3 is widespread in CRC, lung adenocarcinoma, etc. (Zhang et al., 2018). Although pyroptosis can inhibit tumor development, it is pro-inflammatory, creating a microenvironment suitable for tumor growth and thus promoting tumor growth (Xia et al., 2019). Therefore, we focused our efforts on exploring the interactive patterns of pyroptosis in COAD and developed a prognostic predictive signature.

We identified 15 pyroptosis-related lncRNAs in this study, and some of them were already reported to exist in COAD or other cancers. Among them, ZNF667-AS1, OIP5-AS1, AL118506.1, AF117829.1, POC1B-AS1, CCDC18-AS1, THUMPD3-AS1 and FLNB-AS1 were risk-related lncRNAs, while SNHG11, HCG18, AL021707.2, UGDH-AS1, LINC00641, FGD5-AS1 and AC245452.1 were protective lncRNAs. ZNF667-AS1 (lncRNA mortal obligate RNA transcript) plays an important role in gynecological cancers and might be a potential diagnostic marker (Di Fiore et al., 2021). OIP5-AS1 (OIP5 antisense transcript 1), highly expressed in the nervous system and involved in the regulation of the cell cycle, is closely associated with neoplastic transformation (Ghafouri-Fard et al., 2021). In bone metastatic melanoma, AL118506.1 (antisense to abhydrolase domain containing 16B) participates in the ceRNA network of has-miR-27b-3p/AL118506.1/THBS2, which has great utility in predicting survival and metastasis of melanoma (Huang et al., 2019). In severe aplastic anemia (SAA) patients, the decreased expression of AF117829.1 upregulates the activity of CD8^+^ T lymphocytes, which means lncRNA may be associated with the regulation of the immune microenvironment (Li et al., 2021). POC1B-AS1 is also a risk factor in the prognostic model of endometrial carcinoma (Liu J. et al., 2021). In psoriasis, CCDC18-AS1, regarded as immune-related lncRNA, can be a potential diagnostic biomarker (Fan et al., 2021). THUMPD3-AS1 can regulate self-renewal by ceRNA network of miR-543 and ONECUT2 associated with non-small cell lung cancer (Hu et al., 2019). The expression levels of FLNB-AS1 are positively correlated with the survival probability of breast cancer patients (Zhang et al., 2020). The SNHG11/miR-184/CDC25A ceRNA network is a new biomarker for the diagnosis, treatment and prognosis of gastric cancer (Zhao et al., 2021). A previous study has shown that HCG18 participates in vascular invasion of hepatocellular cancer by regulating immune cells (Zhang et al., 2021). Interestingly, AL021707.2, as a part of ferroptosis-related lncRNAs signature of lung adenocarcinoma, can predict the prognosis of lung adenocarcinoma, which is the same as LINC00641 in glioma (Zheng J. et al., 2021; Zheng Z. et al., 2021). UGDH-AS1 has a prognostic value in esophageal cancer (Liu et al., 2020). The activation of FGD5-AS1 can promote the progression of cervical cancer by regulating BST2 to inhibit macrophage M1 polarization (Liu G. et al., 2021).

For a better assessment of the scientific nature of the model building, we compared our pyroptosis-related lncRNAs signature with that of other cancers. In lung adenocarcinoma, Song et al. constructed a pyroptosis-related lncRNAs signature for prognostic prediction (Song et al., 2021). Their idea of finding the pyroptosis-related lncRNAs to build the model was basically similar to ours. The clinical information was also combined to construct the nomogram, and the tumor microenvironment was analyzed. However, the value of the Pearson correlation coefficient was small, and there were no external datasets to validate their signature. Tang et al. built a pyroptosis-related lncRNAs signature for predicting the prognosis of clear cell renal cell carcinoma (ccRCC) (Tang et al., 2021). They performed a large enrichment analysis to reveal the roles of pyroptosis-related lncRNAs in ccRCC from their biological functions. Still, their study required additional confirmation in external datasets. Prior to our study, the association of pyroptosis with colon cancer was similarly explored. Wei et al. conducted a comprehensive bioinformatics analysis of the role of pyroptosis in the development of colon cancer (Wei et al., 2021). They clustered colon cancer samples according to pyroptosis-related genes and obtained three pyroptosis-related subtypes. Then, weighted gene co-expression network analysis (WGCNA) showed that tumor microenvironment was significantly associated with pyroptosis-related subtypes. Finally, the genes in the module were combined with meaningful clinical features of the multivariate Cox analysis to predict the prognosis of colon cancer patients. These studies gave us much inspiration, on which we further incorporated pyroptosis-related lncRNAs for analysis. Thus, we constructed a risk model not only for pyroptosis-related lncRNAs but also for immune infiltrating cells. Deeper insights revealed the tight link between pyroptosis and colon cancer development as well as the tumor microenvironment.

Up to now, all the pyroptosis-related lncRNAs included in constructing the prognostic signature of COAD have been confirmed. Among them is no shortage of those about ferroptosis and apoptosis, which are other forms of programmed cell death. Furthermore, the interaction between these lncRNAs and the immune microenvironment is close and complicated. Therefore, we have reasons to believe that the lncRNAs prognostic signature associated with pyroptosis in COAD has its feasibility. Indubitably, there are some limitations in this study. The validation set from GEO just verified the prediction efficiency of the risk model based on the 15 pyroptosis-related lncRNAs, without conjunction with clinical information. Fundamental experiments of tumor cell lines and clinical samples are missing. Therefore, more experiments are needed to explore the molecular mechanism between the lncRNAs and pyroptosis in COAD.

## Conclusion

To sum up, the pyroptosis-related lncRNAs signature exhibited its capability of predicting the clinical outcomes of COAD patients and participated in the immune microenvironment cellularity. We hope that our findings will provide a basis for the diagnosis and treatment of COAD in the future.

## Data Availability

Publicly available datasets were analyzed in this study. This data can be found here: https://portal.gdc.cancer.gov/, https://www.ncbi.nlm.nih.gov/geo/.
